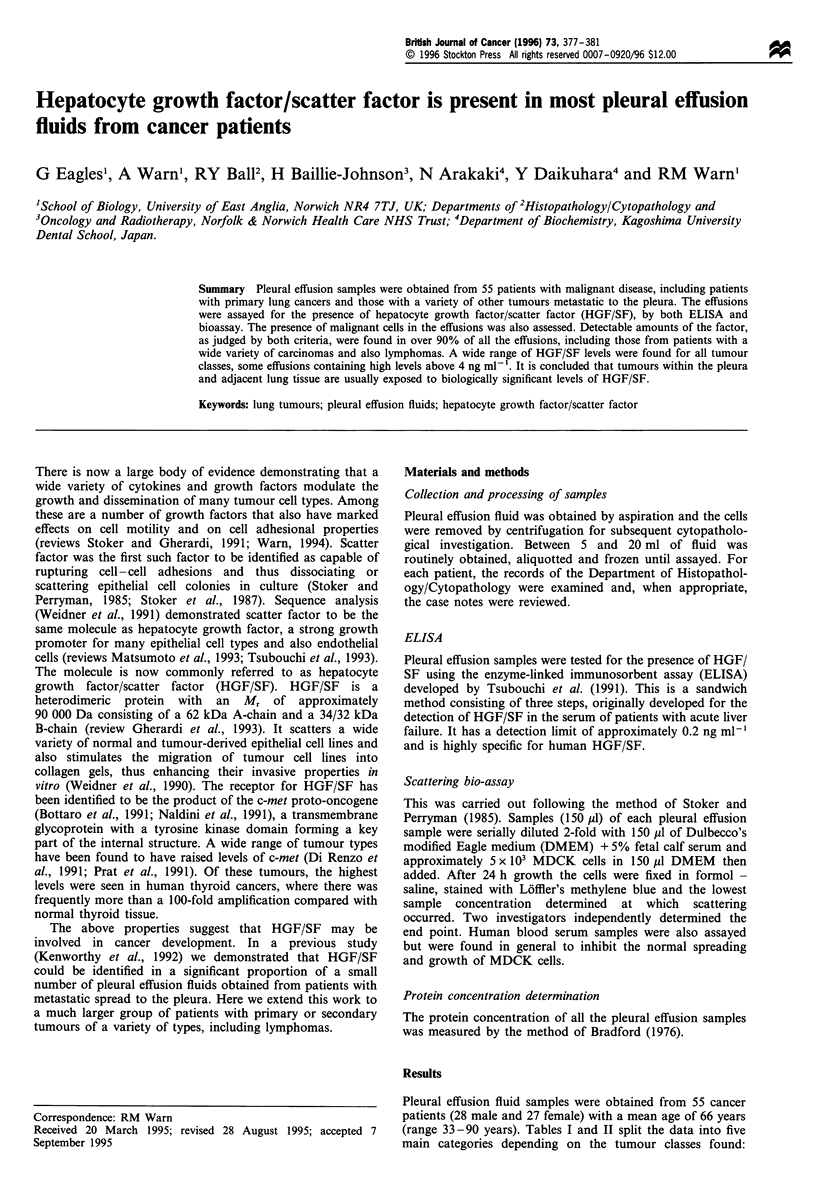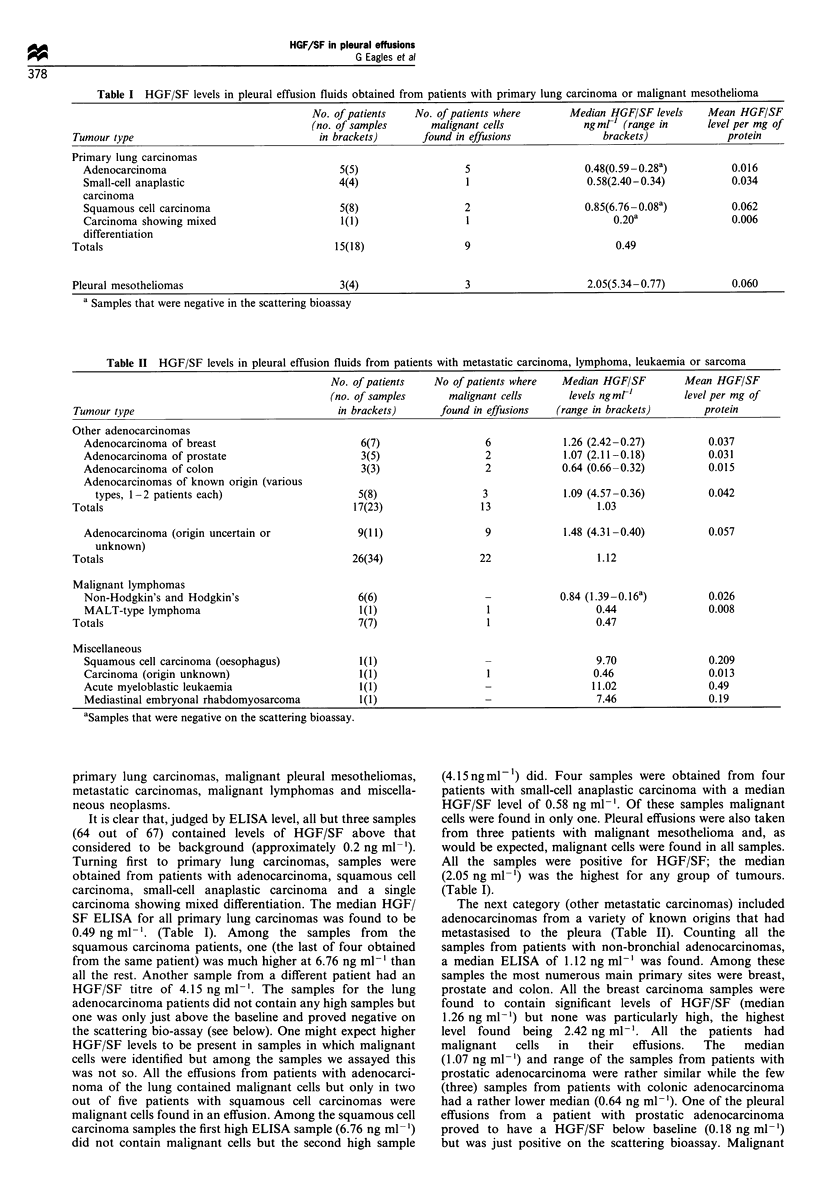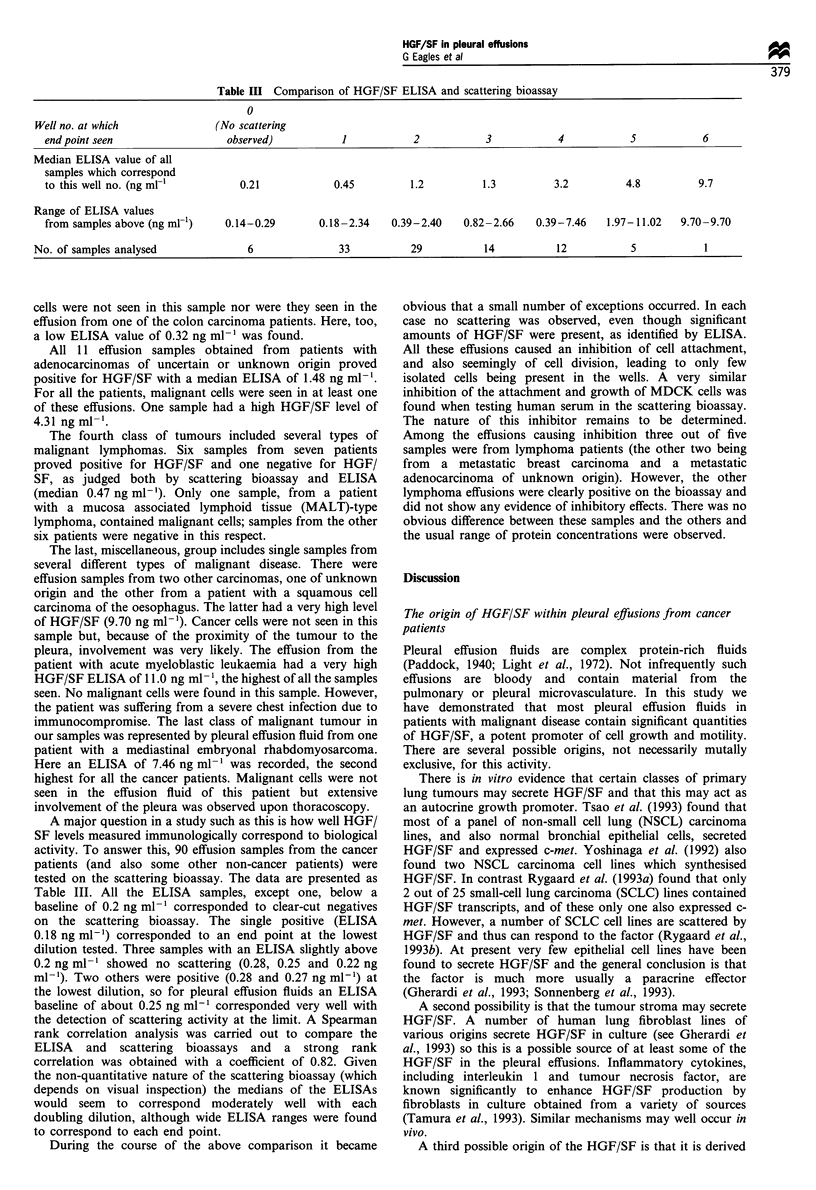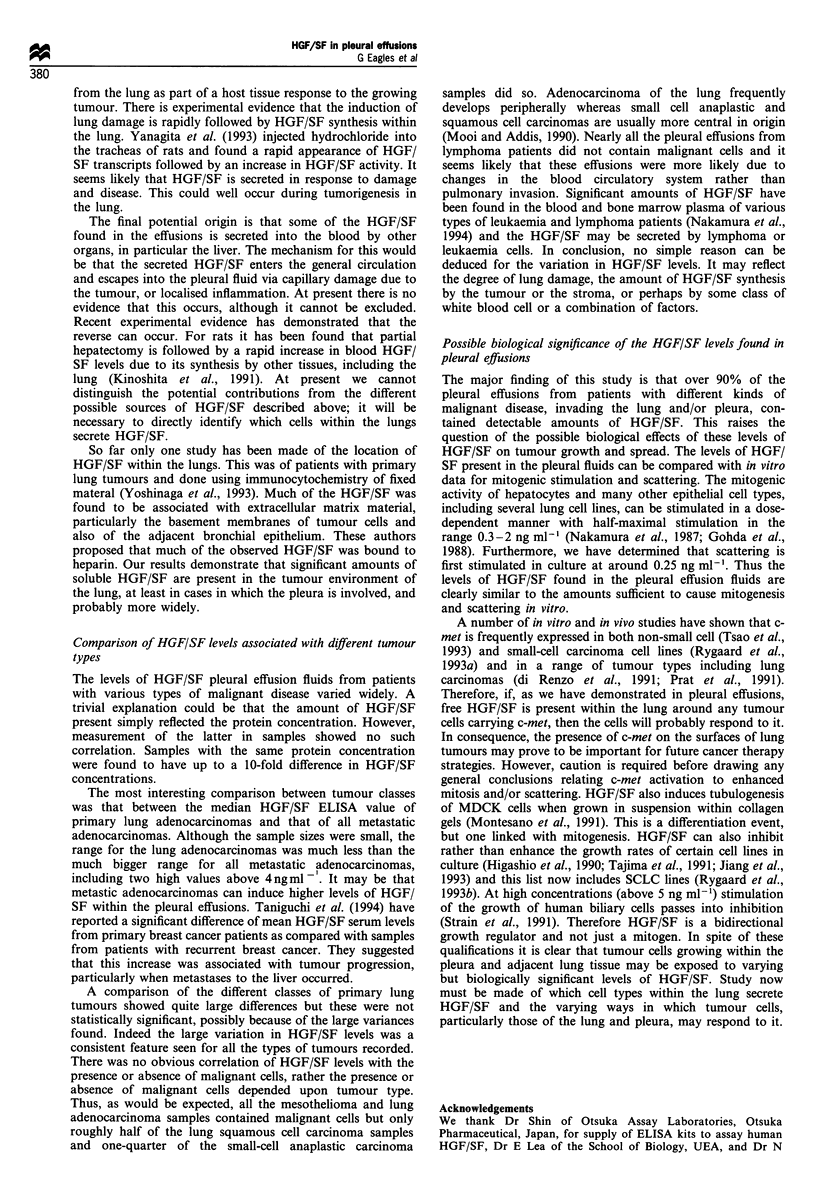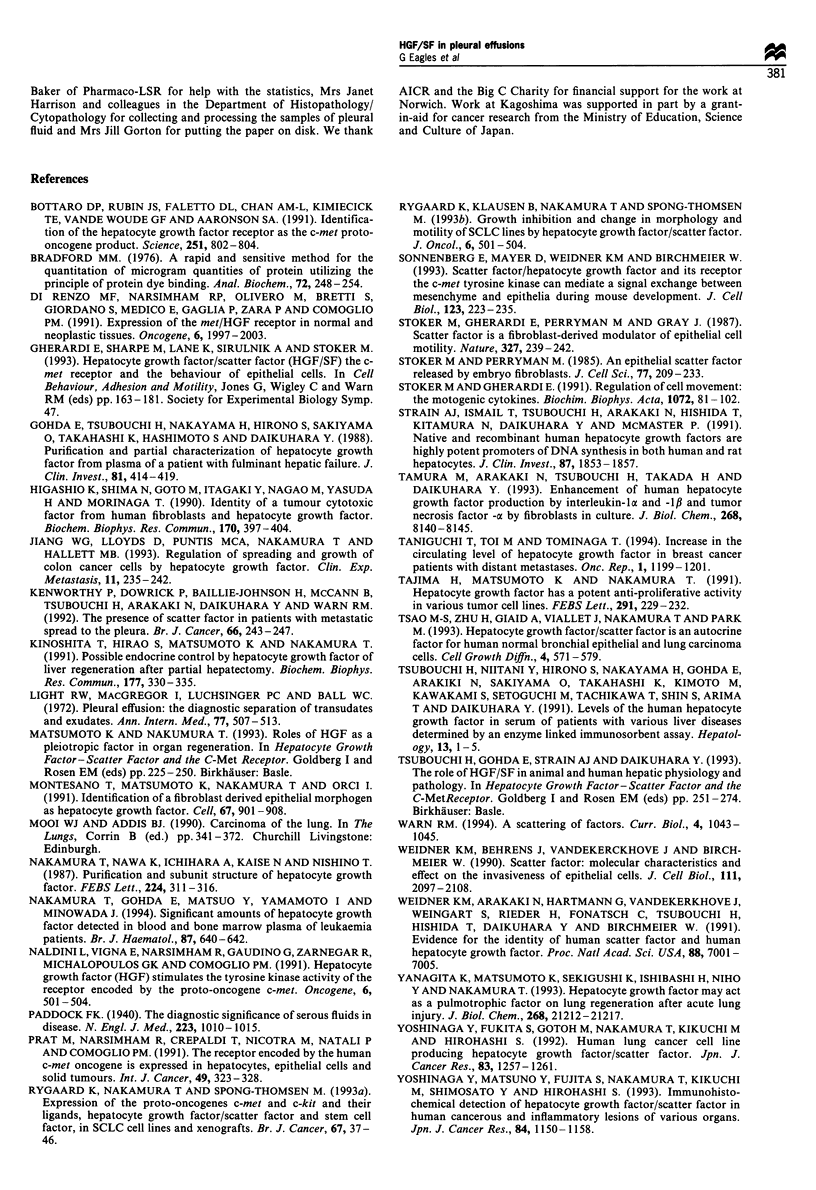# Hepatocyte growth factor/scatter factor is present in most pleural effusion fluids from cancer patients.

**DOI:** 10.1038/bjc.1996.64

**Published:** 1996-02

**Authors:** G. Eagles, A. Warn, R. Y. Ball, H. Baillie-Johnson, N. Arakaki, Y. Daikuhara, R. M. Warn

**Affiliations:** School of Biology, University of East Anglia, Norwich, UK.

## Abstract

Pleural effusion samples were obtained from 55 patients with malignant disease, including patients with primary lung cancers and those with a variety of other tumours metastatic to the pleura. The effusions were assayed for the presence of hepatocyte growth factor/scatter factor (HGF/SF), by both ELISA and bioassay. The presence of malignant cells in the effusions was also assessed. Detectable amounts of the factor, as judged by both criteria, were found in over 90% of all the effusions, including those from patients with a wide variety of carcinomas and also lymphomas. A wide range of HGF/SF levels were found for all tumour classes, some effusions containing high levels above 4 ng ml-1. It is concluded that tumours within the pleura and adjacent lung tissue are usually exposed to biologically significant levels of HGF/SF.


British Journal of Cancer (1996) 73, 377-381
© 1996 Stockton Press All rights reserved 0007-0920/96 $12.00

### Hepatocyte growth factor/scatter factor is present in most pleural effusion fluids from cancer patients

G Eagles[1], A Warn[1], RY Ball[2], H Baillie-Johnson[3], N Arakaki[4], Y Daikuhara[4] and RM Warn[1]

[1]*School of Biology, University of East Anglia, Norwich NR4 7TJ, UK; Departments of* [2]*Histopathology/Cytopathology and* [3]*Oncology and Radiotherapy, Norfolk & Norwich Health Care NHS Trust;* [4]*Department of Biochemistry, Kagoshima University Dental School, Japan.*

**Summary** Pleural effusion samples were obtained from 55 patients with malignant disease, including patients with primary lung cancers and those with a variety of other tumours metastatic to the pleura. The effusions were assayed for the presence of hepatocyte growth factor/scatter factor (HGF/SF), by both ELISA and bioassay. The presence of malignant cells in the effusions was also assessed. Detectable amounts of the factor, as judged by both criteria, were found in over 90% of all the effusions, including those from patients with a wide variety of carcinomas and also lymphomas. A wide range of HGF/SF levels were found for all tumour classes, some effusions containing high levels above 4 ng ml$^{-1}$. It is concluded that tumours within the pleura and adjacent lung tissue are usually exposed to biologically significant levels of HGF/SF.

**Keywords:** lung tumours; pleural effusion fluids; hepatocyte growth factor/scatter factor

There is now a large body of evidence demonstrating that a wide variety of cytokines and growth factors modulate the growth and dissemination of many tumour cell types. Among these are a number of growth factors that also have marked effects on cell motility and on cell adhesional properties (reviews Stoker and Gherardi, 1991; Warn, 1994). Scatter factor was the first such factor to be identified as capable of rupturing cell–cell adhesions and thus dissociating or scattering epithelial cell colonies in culture (Stoker and Perryman, 1985; Stoker *et al.*, 1987). Sequence analysis (Weidner *et al.*, 1991) demonstrated scatter factor to be the same molecule as hepatocyte growth factor, a strong growth promoter for many epithelial cell types and also endothelial cells (reviews Matsumoto *et al.*, 1993; Tsubouchi *et al.*, 1993). The molecule is now commonly referred to as hepatocyte growth factor/scatter factor (HGF/SF). HGF/SF is a heterodimeric protein with an $M_r$ of approximately 90 000 Da consisting of a 62 kDa A-chain and a 34/32 kDa B-chain (review Gherardi *et al.*, 1993). It scatters a wide variety of normal and tumour-derived epithelial cell lines and also stimulates the migration of tumour cell lines into collagen gels, thus enhancing their invasive properties *in vitro* (Weidner *et al.*, 1990). The receptor for HGF/SF has been identified to be the product of the c-*met* proto-oncogene (Bottaro *et al.*, 1991; Naldini *et al.*, 1991), a transmembrane glycoprotein with a tyrosine kinase domain forming a key part of the internal structure. A wide range of tumour types have been found to have raised levels of c-*met* (Di Renzo *et al.*, 1991; Prat *et al.*, 1991). Of these tumours, the highest levels were seen in human thyroid cancers, where there was frequently more than a 100-fold amplification compared with normal thyroid tissue.

The above properties suggest that HGF/SF may be involved in cancer development. In a previous study (Kenworthy *et al.*, 1992) we demonstrated that HGF/SF could be identified in a significant proportion of a small number of pleural effusion fluids obtained from patients with metastatic spread to the pleura. Here we extend this work to a much larger group of patients with primary or secondary tumours of a variety of types, including lymphomas.

Correspondence: RM Warn
Received 20 March 1995; revised 28 August 1995; accepted 7 September 1995

### Materials and methods

### *Collection and processing of samples*

Pleural effusion fluid was obtained by aspiration and the cells were removed by centrifugation for subsequent cytopathological investigation. Between 5 and 20 ml of fluid was routinely obtained, aliquotted and frozen until assayed. For each patient, the records of the Department of Histopathology/Cytopathology were examined and, when appropriate, the case notes were reviewed.

### *ELISA*

Pleural effusion samples were tested for the presence of HGF/SF using the enzyme-linked immunosorbent assay (ELISA) developed by Tsubouchi *et al.* (1991). This is a sandwich method consisting of three steps, originally developed for the detection of HGF/SF in the serum of patients with acute liver failure. It has a detection limit of approximately 0.2 ng ml$^{-1}$ and is highly specific for human HGF/SF.

### *Scattering bio-assay*

This was carried out following the method of Stoker and Perryman (1985). Samples (150 $\mu$l) of each pleural effusion sample were serially diluted 2-fold with 150 $\mu$l of Dulbecco's modified Eagle medium (DMEM) +5% fetal calf serum and approximately $5 \times 10^3$ MDCK cells in 150 $\mu$l DMEM then added. After 24 h growth the cells were fixed in formol - saline, stained with Löffler's methylene blue and the lowest sample concentration determined at which scattering occurred. Two investigators independently determined the end point. Human blood serum samples were also assayed but were found in general to inhibit the normal spreading and growth of MDCK cells.

### *Protein concentration determination*

The protein concentration of all the pleural effusion samples was measured by the method of Bradford (1976).

### Results

Pleural effusion fluid samples were obtained from 55 cancer patients (28 male and 27 female) with a mean age of 66 years (range 33–90 years). Tables I and II split the data into five main categories depending on the tumour classes found:

Table I HGF/SF levels in pleural effusion fluids obtained from patients with primary lung carcinoma or malignant mesothelioma

| Tumour type | No. of patients (no. of samples in brackets) | No. of patients where malignant cells found in effusions | Median HGF/SF levels ng ml$^{-1}$ (range in brackets) | Mean HGF/SF level per mg of protein |
|---|---|---|---|---|
| Primary lung carcinomas | | | | |
| Adenocarcinoma | 5(5) | 5 | 0.48(0.59–0.28[a]) | 0.016 |
| Small-cell anaplastic carcinoma | 4(4) | 1 | 0.58(2.40–0.34) | 0.034 |
| Squamous cell carcinoma | 5(8) | 2 | 0.85(6.76–0.08[a]) | 0.062 |
| Carcinoma showing mixed differentiation | 1(1) | 1 | 0.20[a] | 0.006 |
| Totals | 15(18) | 9 | 0.49 | |
| Pleural mesotheliomas | 3(4) | 3 | 2.05(5.34–0.77) | 0.060 |

[a] Samples that were negative in the scattering bioassay

Table II HGF/SF levels in pleural effusion fluids from patients with metastatic carcinoma, lymphoma, leukaemia or sarcoma

| Tumour type | No. of patients (no. of samples in brackets) | No of patients where malignant cells found in effusions | Median HGF/SF levels ng ml$^{-1}$ (range in brackets) | Mean HGF/SF level per mg of protein |
|---|---|---|---|---|
| Other adenocarcinomas | | | | |
| Adenocarcinoma of breast | 6(7) | 6 | 1.26 (2.42–0.27) | 0.037 |
| Adenocarcinoma of prostate | 3(5) | 2 | 1.07 (2.11–0.18) | 0.031 |
| Adenocarcinoma of colon | 3(3) | 2 | 0.64 (0.66–0.32) | 0.015 |
| Adenocarcinomas of known origin (various types, 1–2 patients each) | 5(8) | 3 | 1.09 (4.57–0.36) | 0.042 |
| Totals | 17(23) | 13 | 1.03 | |
| Adenocarcinoma (origin uncertain or unknown) | 9(11) | 9 | 1.48 (4.31–0.40) | 0.057 |
| Totals | 26(34) | 22 | 1.12 | |
| Malignant lymphomas | | | | |
| Non-Hodgkin's and Hodgkin's | 6(6) | – | 0.84 (1.39–0.16[a]) | 0.026 |
| MALT-type lymphoma | 1(1) | 1 | 0.44 | 0.008 |
| Totals | 7(7) | 1 | 0.47 | |
| Miscellaneous | | | | |
| Squamous cell carcinoma (oesophagus) | 1(1) | – | 9.70 | 0.209 |
| Carcinoma (origin unknown) | 1(1) | 1 | 0.46 | 0.013 |
| Acute myeloblastic leukaemia | 1(1) | – | 11.02 | 0.49 |
| Mediastinal embryonal rhabdomyosarcoma | 1(1) | – | 7.46 | 0.19 |

[a]Samples that were negative on the scattering bioassay.

primary lung carcinomas, malignant pleural mesotheliomas, metastatic carcinomas, malignant lymphomas and miscellaneous neoplasms.

It is clear that, judged by ELISA level, all but three samples (64 out of 67) contained levels of HGF/SF above that considered to be background (approximately 0.2 ng ml$^{-1}$). Turning first to primary lung carcinomas, samples were obtained from patients with adenocarcinoma, squamous cell carcinoma, small-cell anaplastic carcinoma and a single carcinoma showing mixed differentiation. The median HGF/SF ELISA for all primary lung carcinomas was found to be 0.49 ng ml$^{-1}$. (Table I). Among the samples from the squamous carcinoma patients, one (the last of four obtained from the same patient) was much higher at 6.76 ng ml$^{-1}$ than all the rest. Another sample from a different patient had an HGF/SF titre of 4.15 ng ml$^{-1}$. The samples for the lung adenocarcinoma patients did not contain any high samples but one was only just above the baseline and proved negative on the scattering bio-assay (see below). One might expect higher HGF/SF levels to be present in samples in which malignant cells were identified but among the samples we assayed this was not so. All the effusions from patients with adenocarcinoma of the lung contained malignant cells but only in two out of five patients with squamous cell carcinomas were malignant cells found in an effusion. Among the squamous cell carcinoma samples the first high ELISA sample (6.76 ng ml$^{-1}$) did not contain malignant cells but the second high sample (4.15 ng ml$^{-1}$) did. Four samples were obtained from four patients with small-cell anaplastic carcinoma with a median HGF/SF level of 0.58 ng ml$^{-1}$. Of these samples malignant cells were found in only one. Pleural effusions were also taken from three patients with malignant mesothelioma and, as would be expected, malignant cells were found in all samples. All the samples were positive for HGF/SF; the median (2.05 ng ml$^{-1}$) was the highest for any group of tumours. (Table I).

The next category (other metastatic carcinomas) included adenocarcinomas from a variety of known origins that had metastasised to the pleura (Table II). Counting all the samples from patients with non-bronchial adenocarcinomas, a median ELISA of 1.12 ng ml$^{-1}$ was found. Among these samples the most numerous main primary sites were breast, prostate and colon. All the breast carcinoma samples were found to contain significant levels of HGF/SF (median 1.26 ng ml$^{-1}$) but none was particularly high, the highest level found being 2.42 ng ml$^{-1}$. All the patients had malignant cells in their effusions. The median (1.07 ng ml$^{-1}$) and range of the samples from patients with prostatic adenocarcinoma were rather similar while the few (three) samples from patients with colonic adenocarcinoma had a rather lower median (0.64 ng ml$^{-1}$). One of the pleural effusions from a patient with prostatic adenocarcinoma proved to have a HGF/SF below baseline (0.18 ng ml$^{-1}$) but was just positive on the scattering bioassay. Malignant

**Table III** Comparison of HGF/SF ELISA and scattering bioassay

| *Well no. at which end point seen* | *0 (No scattering observed)* | *1* | *2* | *3* | *4* | *5* | *6* |
|---|---|---|---|---|---|---|---|
| Median ELISA value of all samples which correspond to this well no. (ng ml$^{-1}$ | 0.21 | 0.45 | 1.2 | 1.3 | 3.2 | 4.8 | 9.7 |
| Range of ELISA values from samples above (ng ml$^{-1}$) | 0.14–0.29 | 0.18–2.34 | 0.39–2.40 | 0.82–2.66 | 0.39–7.46 | 1.97–11.02 | 9.70–9.70 |
| No. of samples analysed | 6 | 33 | 29 | 14 | 12 | 5 | 1 |

cells were not seen in this sample nor were they seen in the effusion from one of the colon carcinoma patients. Here, too, a low ELISA value of 0.32 ng ml$^{-1}$ was found.

All 11 effusion samples obtained from patients with adenocarcinomas of uncertain or unknown origin proved positive for HGF/SF with a median ELISA of 1.48 ng ml$^{-1}$. For all the patients, malignant cells were seen in at least one of these effusions. One sample had a high HGF/SF level of 4.31 ng ml$^{-1}$.

The fourth class of tumours included several types of malignant lymphomas. Six samples from seven patients proved positive for HGF/SF and one negative for HGF/SF, as judged both by scattering bioassay and ELISA (median 0.47 ng ml$^{-1}$). Only one sample, from a patient with a mucosa associated lymphoid tissue (MALT)-type lymphoma, contained malignant cells; samples from the other six patients were negative in this respect.

The last, miscellaneous, group includes single samples from several different types of malignant disease. There were effusion samples from two other carcinomas, one of unknown origin and the other from a patient with a squamous cell carcinoma of the oesophagus. The latter had a very high level of HGF/SF (9.70 ng ml$^{-1}$). Cancer cells were not seen in this sample but, because of the proximity of the tumour to the pleura, involvement was very likely. The effusion from the patient with acute myeloblastic leukaemia had a very high HGF/SF ELISA of 11.0 ng ml$^{-1}$, the highest of all the samples seen. No malignant cells were found in this sample. However, the patient was suffering from a severe chest infection due to immunocompromise. The last class of malignant tumour in our samples was represented by pleural effusion fluid from one patient with a mediastinal embryonal rhabdomyosarcoma. Here an ELISA of 7.46 ng ml$^{-1}$ was recorded, the second highest for all the cancer patients. Malignant cells were not seen in the effusion fluid of this patient but extensive involvement of the pleura was observed upon thoracoscopy.

A major question in a study such as this is how well HGF/SF levels measured immunologically correspond to biological activity. To answer this, 90 effusion samples from the cancer patients (and also some other non-cancer patients) were tested on the scattering bioassay. The data are presented as Table III. All the ELISA samples, except one, below a baseline of 0.2 ng ml$^{-1}$ corresponded to clear-cut negatives on the scattering bioassay. The single positive (ELISA 0.18 ng ml$^{-1}$) corresponded to an end point at the lowest dilution tested. Three samples with an ELISA slightly above 0.2 ng ml$^{-1}$ showed no scattering (0.28, 0.25 and 0.22 ng ml$^{-1}$). Two others were positive (0.28 and 0.27 ng ml$^{-1}$) at the lowest dilution, so for pleural effusion fluids an ELISA baseline of about 0.25 ng ml$^{-1}$ corresponded very well with the detection of scattering activity at the limit. A Spearman rank correlation analysis was carried out to compare the ELISA and scattering bioassays and a strong rank correlation was obtained with a coefficient of 0.82. Given the non-quantitative nature of the scattering bioassay (which depends on visual inspection) the medians of the ELISAs would seem to correspond moderately well with each doubling dilution, although wide ELISA ranges were found to correspond to each end point.

During the course of the above comparison it became obvious that a small number of exceptions occurred. In each case no scattering was observed, even though significant amounts of HGF/SF were present, as identified by ELISA. All these effusions caused an inhibition of cell attachment, and also seemingly of cell division, leading to only few isolated cells being present in the wells. A very similar inhibition of the attachment and growth of MDCK cells was found when testing human serum in the scattering bioassay. The nature of this inhibitor remains to be determined. Among the effusions causing inhibition three out of five samples were from lymphoma patients (the other two being from a metastatic breast carcinoma and a metastatic adenocarcinoma of unknown origin). However, the other lymphoma effusions were clearly positive on the bioassay and did not show any evidence of inhibitory effects. There was no obvious difference between these samples and the others and the usual range of protein concentrations were observed.

### Discussion

### *The origin of HGF/SF within pleural effusions from cancer patients*

Pleural effusion fluids are complex protein-rich fluids (Paddock, 1940; Light *et al.*, 1972). Not infrequently such effusions are bloody and contain material from the pulmonary or pleural microvasculature. In this study we have demonstrated that most pleural effusion fluids in patients with malignant disease contain significant quantities of HGF/SF, a potent promoter of cell growth and motility. There are several possible origins, not necessarily mutally exclusive, for this activity.

There is *in vitro* evidence that certain classes of primary lung tumours may secrete HGF/SF and that this may act as an autocrine growth promoter. Tsao *et al.* (1993) found that most of a panel of non-small cell lung (NSCL) carcinoma lines, and also normal bronchial epithelial cells, secreted HGF/SF and expressed c-*met*. Yoshinaga *et al.* (1992) also found two NSCL carcinoma cell lines which synthesised HGF/SF. In contrast Rygaard *et al.* (1993*a*) found that only 2 out of 25 small-cell lung carcinoma (SCLC) lines contained HGF/SF transcripts, and of these only one also expressed c-*met*. However, a number of SCLC cell lines are scattered by HGF/SF and thus can respond to the factor (Rygaard *et al.*, 1993*b*). At present very few epithelial cell lines have been found to secrete HGF/SF and the general conclusion is that the factor is much more usually a paracrine effector (Gherardi *et al.*, 1993; Sonnenberg *et al.*, 1993).

A second possibility is that the tumour stroma may secrete HGF/SF. A number of human lung fibroblast lines of various origins secrete HGF/SF in culture (see Gherardi *et al.*, 1993) so this is a possible source of at least some of the HGF/SF in the pleural effusions. Inflammatory cytokines, including interleukin 1 and tumour necrosis factor, are known significantly to enhance HGF/SF production by fibroblasts in culture obtained from a variety of sources (Tamura *et al.*, 1993). Similar mechanisms may well occur *in vivo*.

A third possible origin of the HGF/SF is that it is derived from the lung as part of a host tissue response to the growing tumour. There is experimental evidence that the induction of lung damage is rapidly followed by HGF/SF synthesis within the lung. Yanagita *et al.* (1993) injected hydrochloride into the tracheas of rats and found a rapid appearance of HGF/SF transcripts followed by an increase in HGF/SF activity. It seems likely that HGF/SF is secreted in response to damage and disease. This could well occur during tumorigenesis in the lung.

The final potential origin is that some of the HGF/SF found in the effusions is secreted into the blood by other organs, in particular the liver. The mechanism for this would be that the secreted HGF/SF enters the general circulation and escapes into the pleural fluid via capillary damage due to the tumour, or localised inflammation. At present there is no evidence that this occurs, although it cannot be excluded. Recent experimental evidence has demonstrated that the reverse can occur. For rats it has been found that partial hepatectomy is followed by a rapid increase in blood HGF/SF levels due to its synthesis by other tissues, including the lung (Kinoshita *et al.*, 1991). At present we cannot distinguish the potential contributions from the different possible sources of HGF/SF described above; it will be necessary to directly identify which cells within the lungs secrete HGF/SF.

So far only one study has been made of the location of HGF/SF within the lungs. This was of patients with primary lung tumours and done using immunocytochemistry of fixed materal (Yoshinaga *et al.*, 1993). Much of the HGF/SF was found to be associated with extracellular matrix material, particularly the basement membranes of tumour cells and also of the adjacent bronchial epithelium. These authors proposed that much of the observed HGF/SF was bound to heparin. Our results demonstrate that significant amounts of soluble HGF/SF are present in the tumour environment of the lung, at least in cases in which the pleura is involved, and probably more widely.

*Comparison of HGF/SF levels associated with different tumour types*

The levels of HGF/SF pleural effusion fluids from patients with various types of malignant disease varied widely. A trivial explanation could be that the amount of HGF/SF present simply reflected the protein concentration. However, measurement of the latter in samples showed no such correlation. Samples with the same protein concentration were found to have up to a 10-fold difference in HGF/SF concentrations.

The most interesting comparison between tumour classes was that between the median HGF/SF ELISA value of primary lung adenocarcinomas and that of all metastatic adenocarcinomas. Although the sample sizes were small, the range for the lung adenocarcinomas was much less than the much bigger range for all metastatic adenocarcinomas, including two high values above 4 ng ml$^{-1}$. It may be that metastic adenocarcinomas can induce higher levels of HGF/SF within the pleural effusions. Taniguchi *et al.* (1994) have reported a significant difference of mean HGF/SF serum levels from primary breast cancer patients as compared with samples from patients with recurrent breast cancer. They suggested that this increase was associated with tumour progression, particularly when metastases to the liver occurred.

A comparison of the different classes of primary lung tumours showed quite large differences but these were not statistically significant, possibly because of the large variances found. Indeed the large variation in HGF/SF levels was a consistent feature seen for all the types of tumours recorded. There was no obvious correlation of HGF/SF levels with the presence or absence of malignant cells, rather the presence or absence of malignant cells depended upon tumour type. Thus, as would be expected, all the mesothelioma and lung adenocarcinoma samples contained malignant cells but only roughly half of the lung squamous cell carcinoma samples and one-quarter of the small-cell anaplastic carcinoma samples did so. Adenocarcinoma of the lung frequently develops peripherally whereas small cell anaplastic and squamous cell carcinomas are usually more central in origin (Mooi and Addis, 1990). Nearly all the pleural effusions from lymphoma patients did not contain malignant cells and it seems likely that these effusions were more likely due to changes in the blood circulatory system rather than pulmonary invasion. Significant amounts of HGF/SF have been found in the blood and bone marrow plasma of various types of leukaemia and lymphoma patients (Nakamura *et al.*, 1994) and the HGF/SF may be secreted by lymphoma or leukaemia cells. In conclusion, no simple reason can be deduced for the variation in HGF/SF levels. It may reflect the degree of lung damage, the amount of HGF/SF synthesis by the tumour or the stroma, or perhaps by some class of white blood cell or a combination of factors.

*Possible biological significance of the HGF/SF levels found in pleural effusions*

The major finding of this study is that over 90% of the pleural effusions from patients with different kinds of malignant disease, invading the lung and/or pleura, contained detectable amounts of HGF/SF. This raises the question of the possible biological effects of these levels of HGF/SF on tumour growth and spread. The levels of HGF/SF present in the pleural fluids can be compared with *in vitro* data for mitogenic stimulation and scattering. The mitogenic activity of hepatocytes and many other epithelial cell types, including several lung cell lines, can be stimulated in a dose-dependent manner with half-maximal stimulation in the range 0.3–2 ng ml$^{-1}$ (Nakamura *et al.*, 1987; Gohda *et al.*, 1988). Furthermore, we have determined that scattering is first stimulated in culture at around 0.25 ng ml$^{-1}$. Thus the levels of HGF/SF found in the pleural effusion fluids are clearly similar to the amounts sufficient to cause mitogenesis and scattering *in vitro*.

A number of *in vitro* and *in vivo* studies have shown that c-*met* is frequently expressed in both non-small cell (Tsao *et al.*, 1993) and small-cell carcinoma cell lines (Rygaard *et al.*, 1993*a*) and in a range of tumour types including lung carcinomas (di Renzo *et al.*, 1991; Prat *et al.*, 1991). Therefore, if, as we have demonstrated in pleural effusions, free HGF/SF is present within the lung around any tumour cells carrying c-*met*, then the cells will probably respond to it. In consequence, the presence of c-*met* on the surfaces of lung tumours may prove to be important for future cancer therapy strategies. However, caution is required before drawing any general conclusions relating c-*met* activation to enhanced mitosis and/or scattering. HGF/SF also induces tubulogenesis of MDCK cells when grown in suspension within collagen gels (Montesano *et al.*, 1991). This is a differentiation event, but one linked with mitogenesis. HGF/SF can also inhibit rather than enhance the growth rates of certain cell lines in culture (Higashio *et al.*, 1990; Tajima *et al.*, 1991; Jiang *et al.*, 1993) and this list now includes SCLC lines (Rygaard *et al.*, 1993*b*). At high concentrations (above 5 ng ml$^{-1}$) stimulation of the growth of human biliary cells passes into inhibition (Strain *et al.*, 1991). Therefore HGF/SF is a bidirectional growth regulator and not just a mitogen. In spite of these qualifications it is clear that tumour cells growing within the pleura and adjacent lung tissue may be exposed to varying but biologically significant levels of HGF/SF. Study now must be made of which cell types within the lung secrete HGF/SF and the varying ways in which tumour cells, particularly those of the lung and pleura, may respond to it.

**Acknowledgements**
We thank Dr Shin of Otsuka Assay Laboratories, Otsuka Pharmaceutical, Japan, for supply of ELISA kits to assay human HGF/SF, Dr E Lea of the School of Biology, UEA, and Dr N

Baker of Pharmaco-LSR for help with the statistics, Mrs Janet Harrison and colleagues in the Department of Histopathology/Cytopathology for collecting and processing the samples of pleural fluid and Mrs Jill Gorton for putting the paper on disk. We thank AICR and the Big C Charity for financial support for the work at Norwich. Work at Kagoshima was supported in part by a grant-in-aid for cancer research from the Ministry of Education, Science and Culture of Japan.

### References

BOTTARO DP, RUBIN JS, FALETTO DL, CHAN AM-L, KIMIECICK TE, VANDE WOUDE GF AND AARONSON SA. (1991). Identification of the hepatocyte growth factor receptor as the c-*met* proto-oncogene product. *Science*, **251**, 802–804.

BRADFORD MM. (1976). A rapid and sensitive method for the quantitation of microgram quantities of protein utilizing the principle of protein dye binding. *Anal. Biochem.*, **72**, 248–254.

DI RENZO MF, NARSIMHAM RP, OLIVERO M, BRETTI S, GIORDANO S, MEDICO E, GAGLIA P, ZARA P AND COMOGLIO PM. (1991). Expression of the *met*/HGF receptor in normal and neoplastic tissues. *Oncogene*, **6**, 1997–2003.

GHERARDI E, SHARPE M, LANE K, SIRULNIK A AND STOKER M. (1993). Hepatocyte growth factor/scatter factor (HGF/SF) the c-*met* receptor and the behaviour of epithelial cells. In *Cell Behaviour, Adhesion and Motility*, Jones G, Wigley C and Warn RM (eds) pp. 163–181. Society for Experimental Biology Symp. 47.

GOHDA E, TSUBOUCHI H, NAKAYAMA H, HIRONO S, SAKIYAMA O, TAKAHASHI K, HASHIMOTO S AND DAIKUHARA Y. (1988). Purification and partial characterization of hepatocyte growth factor from plasma of a patient with fulminant hepatic failure. *J. Clin. Invest.*, **81**, 414–419.

HIGASHIO K, SHIMA N, GOTO M, ITAGAKI Y, NAGAO M, YASUDA H AND MORINAGA T. (1990). Identity of a tumour cytotoxic factor from human fibroblasts and hepatocyte growth factor. *Biochem. Biophys. Res. Commun.*, **170**, 397–404.

JIANG WG, LLOYDS D, PUNTIS MCA, NAKAMURA T AND HALLETT MB. (1993). Regulation of spreading and growth of colon cancer cells by hepatocyte growth factor. *Clin. Exp. Metastasis*, **11**, 235–242.

KENWORTHY P, DOWRICK P, BAILLIE-JOHNSON H, McCANN B, TSUBOUCHI H, ARAKAKI N, DAIKUHARA Y AND WARN RM. (1992). The presence of scatter factor in patients with metastatic spread to the pleura. *Br. J. Cancer*, **66**, 243–247.

KINOSHITA T, HIRAO S, MATSUMOTO K AND NAKAMURA T. (1991). Possible endocrine control by hepatocyte growth factor of liver regeneration after partial hepatectomy. *Biochem. Biophys. Res. Commun.*, **177**, 330–335.

LIGHT RW, MACGREGOR I, LUCHSINGER PC AND BALL WC. (1972). Pleural effusion: the diagnostic separation of transudates and exudates. *Ann. Intern. Med.*, **77**, 507–513.

MATSUMOTO K AND NAKUMURA T. (1993). Roles of HGF as a pleiotropic factor in organ regeneration. In *Hepatocyte Growth Factor–Scatter Factor and the C*-Met *Receptor*. Goldberg I and Rosen EM (eds) pp. 225–250. Birkhäuser: Basle.

MONTESANO T, MATSUMOTO K, NAKAMURA T AND ORCI I. (1991). Identification of a fibroblast derived epithelial morphogen as hepatocyte growth factor. *Cell*, **67**, 901–908.

MOOI WJ AND ADDIS BJ. (1990). Carcinoma of the lung. In *The Lungs*, Corrin B (ed.) pp. 341–372. Churchill Livingstone: Edinburgh.

NAKAMURA T, NAWA K, ICHIHARA A, KAISE N AND NISHINO T. (1987). Purification and subunit structure of hepatocyte growth factor. *FEBS Lett.*, **224**, 311–316.

NAKAMURA T, GOHDA E, MATSUO Y, YAMAMOTO I AND MINOWADA J. (1994). Significant amounts of hepatocyte growth factor detected in blood and bone marrow plasma of leukaemia patients. *Br. J. Haematol.*, **87**, 640–642.

NALDINI L, VIGNA E, NARSIMHAM R, GAUDINO G, ZARNEGAR R, MICHALOPOULOS GK AND COMOGLIO PM. (1991). Hepatocyte growth factor (HGF) stimulates the tyrosine kinase activity of the receptor encoded by the proto-oncogene c-*met*. *Oncogene*, **6**, 501–504.

PADDOCK FK. (1940). The diagnostic significance of serous fluids in disease. *N. Engl. J. Med.*, **223**, 1010–1015.

PRAT M, NARSIMHAM R, CREPALDI T, NICOTRA M, NATALI P AND COMOGLIO PM. (1991). The receptor encoded by the human c-*met* oncogene is expressed in hepatocytes, epithelial cells and solid tumours. *Int. J. Cancer*, **49**, 323–328.

RYGAARD K, NAKAMURA T AND SPONG-THOMSEN M. (1993*a*). Expression of the proto-oncogenes c-*met* and c-*kit* and their ligands, hepatocyte growth factor/scatter factor and stem cell factor, in SCLC cell lines and xenografts. *Br. J. Cancer*, **67**, 37–46.

RYGAARD K, KLAUSEN B, NAKAMURA T AND SPONG-THOMSEN M. (1993*b*). Growth inhibition and change in morphology and motility of SCLC lines by hepatocyte growth factor/scatter factor. *J. Oncol.*, **6**, 501–504.

SONNENBERG E, MAYER D, WEIDNER KM AND BIRCHMEIER W. (1993). Scatter factor/hepatocyte growth factor and its receptor the c-*met* tyrosine kinase can mediate a signal exchange between mesenchyme and epithelia during mouse development. *J. Cell Biol.*, **123**, 223–235.

STOKER M, GHERARDI E, PERRYMAN M AND GRAY J. (1987). Scatter factor is a fibroblast-derived modulator of epithelial cell motility. *Nature*, **327**, 239–242.

STOKER M AND PERRYMAN M. (1985). An epithelial scatter factor released by embryo fibroblasts. *J. Cell Sci.*, **77**, 209–233.

STOKER M AND GHERARDI E. (1991). Regulation of cell movement: the motogenic cytokines. *Biochim. Biophys. Acta*, **1072**, 81–102.

STRAIN AJ, ISMAIL T, TSUBOUCHI H, ARAKAKI N, HISHIDA T, KITAMURA N, DAIKUHARA Y AND McMASTER P. (1991). Native and recombinant human hepatocyte growth factors are highly potent promoters of DNA synthesis in both human and rat hepatocytes. *J. Clin. Invest.*, **87**, 1853–1857.

TAMURA M, ARAKAKI N, TSUBOUCHI H, TAKADA H AND DAIKUHARA Y. (1993). Enhancement of human hepatocyte growth factor production by interleukin-1$\alpha$ and -1$\beta$ and tumor necrosis factor -$\alpha$ by fibroblasts in culture. *J. Biol. Chem.*, **268**, 8140–8145.

TANIGUCHI T, TOI M AND TOMINAGA T. (1994). Increase in the circulating level of hepatocyte growth factor in breast cancer patients with distant metastases. *Onc. Rep.*, **1**, 1199–1201.

TAJIMA H, MATSUMOTO K AND NAKAMURA T. (1991). Hepatocyte growth factor has a potent anti-proliferative activity in various tumor cell lines. *FEBS Lett.*, **291**, 229–232.

TSAO M-S, ZHU H, GIAID A, VIALLET J, NAKAMURA T AND PARK M. (1993). Hepatocyte growth factor/scatter factor is an autocrine factor for human normal bronchial epithelial and lung carcinoma cells. *Cell Growth Diffn.*, **4**, 571–579.

TSUBOUCHI H, NIITANI Y, HIRONO S, NAKAYAMA H, GOHDA E, ARAKIKI N, SAKIYAMA O, TAKAHASHI K, KIMOTO M, KAWAKAMI S, SETOGUCHI M, TACHIKAWA T, SHIN S, ARIMA T AND DAIKUHARA Y. (1991). Levels of the human hepatocyte growth factor in serum of patients with various liver diseases determined by an enzyme linked immunosorbent assay. *Hepatology*, **13**, 1–5.

TSUBOUCHI H, GOHDA E, STRAIN AJ AND DAIKUHARA Y. (1993). The role of HGF/SF in animal and human hepatic physiology and pathology. In *Hepatocyte Growth Factor–Scatter Factor and the C*-Met*Receptor*. Goldberg I and Rosen EM (eds) pp. 251–274. Birkhäuser: Basle.

WARN RM. (1994). A scattering of factors. *Curr. Biol.*, **4**, 1043–1045.

WEIDNER KM, BEHRENS J, VANDEKERCKHOVE J AND BIRCHMEIER W. (1990). Scatter factor: molecular characteristics and effect on the invasiveness of epithelial cells. *J. Cell Biol.*, **111**, 2097–2108.

WEIDNER KM, ARAKAKI N, HARTMANN G, VANDEKERKHOVE J, WEINGART S, RIEDER H, FONATSCH C, TSUBOUCHI H, HISHIDA T, DAIKUHARA Y AND BIRCHMEIER W. (1991). Evidence for the identity of human scatter factor and human hepatocyte growth factor. *Proc. Natl Acad. Sci. USA*, **88**, 7001–7005.

YANAGITA K, MATSUMOTO K, SEKIGUSHI K, ISHIBASHI H, NIHO Y AND NAKAMURA T. (1993). Hepatocyte growth factor may act as a pulmotrophic factor on lung regeneration after acute lung injury. *J. Biol. Chem.*, **268**, 21212–21217.

YOSHINAGA Y, FUKITA S, GOTOH M, NAKAMURA T, KIKUCHI M AND HIROHASHI S. (1992). Human lung cancer cell line producing hepatocyte growth factor/scatter factor. *Jpn. J. Cancer Res.*, **83**, 1257–1261.

YOSHINAGA Y, MATSUNO Y, FUJITA S, NAKAMURA T, KIKUCHI M, SHIMOSATO Y AND HIROHASHI S. (1993). Immunohistochemical detection of hepatocyte growth factor/scatter factor in human cancerous and inflammatory lesions of various organs. *Jpn. J. Cancer Res.*, **84**, 1150–1158.